# Expression of Glutathione Peroxidase and Glutathione Reductase and Level of Free Radical Processes under Toxic Hepatitis in Rats

**DOI:** 10.1155/2013/870628

**Published:** 2013-03-11

**Authors:** Igor Y. Iskusnykh, Tatyana N. Popova, Aleksander A. Agarkov, Miguel Â. A. Pinheiro de Carvalho, Stanislav G. Rjevskiy

**Affiliations:** ^1^Department of Medical Biochemistry and Microbiology, Voronezh State University, University Square 1, Voronezh 394006, Russia; ^2^ISOPlexis Gene Bank, University of Madeira, Campus da Penteada, 9000-390 Funchal, Portugal

## Abstract

Correlation between intensity of free radical processes estimated by biochemiluminesce parameters, content of lipoperoxidation products, and changes of glutathione peroxidase (GP, EC 1.11.1.9) and glutathione reductase (GR, EC 1.6.4.2) activities at rats liver injury, after 12, 36, 70, 96, 110, and 125 hours & tetrachloromethane administration have been investigated. The histological examination of the liver sections of rats showed that prominent hepatocytes with marked vacuolisation and inflammatory cells which were arranged around the necrotic tissue are more at 96 h after exposure to CCl_4_. Moreover maximum increase in GR and GP activities, 2.1 and 2.5 times, respectively, was observed at 96 h after exposure to CCl_4_, what coincided with the maximum of free radical oxidation processes. Using a combination of reverse transcription and real-time polymerase chain reaction, expression of the glutathione peroxidase and glutathione reductase genes (Gpx1 and Gsr) was analyzed by the determination of their respective mRNAs in the rat liver tissue under toxic hepatitis conditions. The analyses of Gpx1 and Gsr expression revealed that the transcript levels increased in 2.5- and 3.0-folds, respectively. Western blot analysis revealed that the amounts of hepatic Gpx1 and Gsr proteins increased considerably after CCl_4_ administration. It can be proposed that the overexpression of these enzymes could be a mechanism of enhancement of hepatocytes tolerance to oxidative stress.

## 1. Introduction

Toxic hepatitis is one of the most widespread pathology of the liver among clinic diseases of internal organs. The accumulation of different xenobiotics can promote development of the disease. High hepatotropic effect is attributed to tetrachloromethane (CCl_4_), which its biotransformation is associated with the functioning of cytochrome P450-dependent monooxygenase system and free radicals production [1]. This process is associated with disruption of the antioxidant protection system of the liver tissue and ultimately leads to an oxidative stress [2, 3]. 

Free radical (FR) processes play an extremely important role in cell activity. However, the intensification of free radical oxidation (FRO) is a key mechanism involved in cellular pathology and apoptosis. The FRO processes are controlled by the antioxidant system (AOS). In case of superfluous generation of reactive oxygen species (ROS) resulting from ionizing radiation, infectious agents, toxins, ischemia, and other pathological factors, the FR process becomes a cascade of events that leads to lipid-lipid and protein-lipid disorders, uncoupling the oxidative phosphorylation and tissue respiration, and in consequence results in a severe imbalance of cellular metabolism.

Protection against the damaging effects of free radicals is carried out by nonenzymatic and enzymatic antioxidant system chains, whereas an important place belongs to the glutathione system. In this system, glutathione peroxidase (GP, EC 1.11.1.9) provides detoxification of organic and inorganic peroxides by using reduced glutathione (GSH). The regeneration of oxidized glutathione (GS) is carried out by glutathione reductase (GR, EC 1.6.4.2), which uses NADPH as reduced equivalents [4, 5]. In this context, the comparative analysis of the temporal dynamics of FRO activity and the expression of GR and GP during the experimental toxic hepatitis could assist in understanding of the mechanisms of glutathione system regulation in rat liver.

## 2. Experimental Details

Male Wistar rats, weighting 150–200 g, were used in the modulation of experimental toxic hepatitis. All experimental procedures were performed according to the international rules for humane treatment of laboratory animals and in accordance with the sanitary rules for maintenance of experimental biological clinics (vivarium). The animals were divided into two experimental groups: (1) control (*n* = 12); (2) animals with experimental toxic hepatitis (*n* = 12).

Tetrachloromethane (CCl_4_), an organ-specific toxin with hepatotropic action was used to simulate an experimental toxic hepatitis. CCl_4_ was administered orally once after one-day food deprivation at a dose of 0.064 mL/100 g of live weight as a solution in Vaseline oil [6–9]. Slaughter of animals was performed after 12, 36, 70, 96, 110, and 125 hours after tetrachloromethane administration.

For histological examination the liver tissues were removed from each group of rats and fixed in 10% formaldehyde at 4°C for 2 days. Fixed specimens were dehydrated and embedded in paraffin. The specimens were then cut into 5 *μ*m thick sections at 50 *μ*m intervals and then stained with hematoxylin and eosin. The sections were then observed with a light microscope.

For determination of intensity of free radical processes at liver and serum of rats of experimental group, the method of biochemiluminescence induced by hydrogen peroxide with iron sulfate was used. The method is based on the fact that in the presented system the catalytic decomposition of hydrogen peroxide by metal ions with variable valence (Fe^2+^) at Fenton reaction takes place. The resulting free radicals are initiated free radical processes in the biological system. Recombination of radicals RO_2_^•^ leads to the formation of an unstable tetroxide, which decays, emitting a photon. The proceeding process was recorded during 30 s. Indicators of the process are light sum of chemiluminescence (*S*), maximum flash intensity (Imax), and the tangent of the angle of the kinetic curve (tg*α*_2_). Mixture for determination of biochemiluminescence intensity had the following composition: 0.4 mL of 0.02 M potassium phosphate buffer (pH 7.5), 0.4 mL of 0.01 mM FeSO_4_, and 0.2 mL of a 2% solution of hydrogen peroxide (introducing directly before measurement). Investigated suspensions were added in amount of 0.1 mL.

With the purpose of enzymes determination activities the liver tissue was homogenized with a T10 homogenizer in fourfold volume of extraction medium (0.1 M Tris-HCl buffer (pH 7.8) containing 1 mM EDTA and 1%  *β*-mercaptoethanol). The homogenate was centrifuged at 1000 g for 15 min. The GP and GR activities were measured spectrophotometrically at 340 nm with a Hitachi-U1900 spectrophotometer. One enzyme unit (U) was defined as the amount of enzyme that catalyzed conversion of 1 *μ*mole of substrate per minute at 25°C. Enzyme activity was expressed in U per gram of live weight of liver, while enzyme specific activity was given as the amount of U per mg of protein. 

GR activity was measured in a reagent mixture composed of 50 mM potassium phosphate buffer (pH 7.4), 1 mM EDTA, 0.16 mM NADPH, and 0.8 mM oxidized glutathione (GSSG).

Measurement of GP activity was performed in 50 mM potassium phosphate buffer (pH 7.4) containing 1 mM EDTA, 0.12 mM NADPH, 0.85 mM GSH, 0.37 mM H_2_O_2_, and 1 U/mL GR. 

To determine the content of diene conjugates spectrophotometric method was used based on the fact that in the course of lipid peroxidation in the stage of formation of free radicals in the molecules of fatty acids, a system of conjugated double bonds. This is accompanied by the appearance of the maximum in the absorption spectrum at 233 nm [10]. 0.25 mL of the test liquid was ground for 15 minutes in a Potter-Elvehjem homogenizer with 9 mL of extraction mixture of heptane with isopropyl alcohol in a volume ratio of 1 : 1. The resulting suspension was placed in a tightly sealed plastic tubes. Samples were centrifuged at 4000 g for 10 minutes. The supernatant was transferred to a graduated test tube, and 1/10 volume of distilled water. Then heptane phase obtained after double shaken and phases separation was used. To an equal volume of 0.5 mL was poured alcohol by volume 1 : 5–1 : 10. The optical density of the samples was determined by spectrophotometry at 233 nm. As a control sample containing only the extracted phase or instead of 1 mL of the supernatant suspension of 1 mL of 0.1 mM phosphate buffer (pH 7,6) was used.

DC content in the homogenate was calculated using the formula: [Diene conjugates] = (*V*_1_∗*D*∗10^6^)/(*L*∗*E*∗*V*_2_) umol/L, where *V*_1_ is the amount of the resulting sample, mL; *D* is the optical density units; *L* is the optical path length, cm; *E* is molar extinction coefficient equal to 2.2∗10^5^ *M* ^−1^ *s* ^−1^; *V*_2_ is introduced by the sample volume, mL.

To assess the expression of Gpx1 and Gsr, the total RNA from liver tissue of control and experimental animals was isolated using a set of YellowSolve (“Clonogene,” Russia). The degree of RNA degradation was evaluated by electrophoresis in denaturing agarose gel (1%). An amount of RNA was measured using a Hitachi-U1900 spectrophotometer at a wavelength of 260 nm. For reverse transcription, 5 micrograms of total RNA was annealed with 1 *μ*L random hexaprimers (0.5 mg/*μ*L) at 70°C for 5 min. After that, the tube was placed in ice and the reagents were added in the following order: 4 *μ*L 5x-RT buffer, 1 *μ*L RiboLock (20 *μ*L/*μ*L), and 2 *μ*L 10 mM dNTP mix. The mixture was incubated at 37°C for 5 min. Then 2 *μ*L of M-MuLV reverse transcriptase (20 units/*μ*L) to a final volume of 20 *μ*L was added. The mixture was subsequently incubated at 37°C for 60 min. The reaction was stopped by heating at 70°C for 10 min. cDNA obtained during reverse transcription was used to analyze gene expression of Gpx1 and Gsr by RT-PCR.

To normalize the expression level of the studied genes, three out group genes were used: albumin (Alb), *β*-actin (*β*-Act), and ribosomal protein L3. Primers for all five investigated genes were selected using Genomics Expression software (Table 1). RT-PCR was carried out using a set of reagents with SYBR Green I (“Synthol” (Russia)) on the apparatus ANA-32 (Research Institute for Analytical Instrumentation RAS, MSTU Russia), as follows: 120 sec at 95°C; whereas at 40 cycles: 15 sec at 95°C; 10 sec, at annealing temperature for the primers of each gene (60°C at for Alb, 63°C at for *β*-Act, 68°C at for ribosomal protein L3, 62°C at for Gsr and Gpx1); and 30 sec at 72°C. Each tissue sample was analyzed three times for each gene. The obtained threshold cycle reactions values were normalized with respect to Ct of three “housekeeping” genes and statistically processed using the “Relative Expression Software Tool (REST)” [11]. The changes of expression relative to control samples were calculated by the following formula: expression = geometric mean (GOI Conc/REFConc1, GOI Conc/2REFConc2…), where GOI Conc is the concentration of the studied genes; REF Conc1, 2… are the concentrations of the reference genes.

Concentration of the studied genes and reference genes was calculated as GOI Conc = efficiency^ΔCt GOI^; REF Conc1, 2…= ΔCt efficiency^REF 1, 2^, where ΔCt GOI is a difference of threshold cycles of control and experimental groups obtained during the study of gene amplification; ΔCt REF Conc1, 2…—a difference of threshold cycles of control and experimental groups obtained during the reference genes amplification.

The amplification efficiency value of the reference and the studied genes was determined using a threshold cycles plot of the cDNA concentration logarithm. For the calculations, the following formula was used: *E* = 10^(−1/*A*)^, where *E* is efficiency of amplification, and *A* is coefficient before argument of linear function.

To improve the accuracy of amplification efficiency in the calculation, the number of repetitions for each dilution was increased with each point of the standard curve representing the mean of measurement for three identical samples. 

After amplification with gene-specific primers the products of PCR reaction were separated by electrophoresis in 1% agarose gel. Fragments of appropriate sizes were cut from the gel and purified using a set of ALMA-Clean Up (Almabion, Russia) according to the protocol of manufacturer. The PCR products were sequenced on an automatic analyzer ABI Prizm 3130 Genetic Analyzer. The obtained sequences were analyzed for specificity using the software BLAST.

After homogenization of the liver from all experimental rats using a T10 homogenizer, the homogenates (50 *μ*g) were separated by sodium dodecyl sulfate polyacrylamide gel electrophoresis, and Western blot analysis was performed using an anti-rat GR antibody, and GP antibody (1 : 1000) purchased from Almabion Co. Ltd. (Russia). The blots were probed with a goat polyclonal actin antibody (1 : 500) from Almabion Co. Ltd (Russia) to ensure that the amount of protein loaded into each lane of the gel was constant. Blots were incubated with the appropriate peroxidase-conjugated secondary antibodies. After removal of the secondary antibodies, the blots were washed and developed using the ECL-Western blotting system.

All data were processed using standard statistical methods [12]. Correlation coefficients between the intensity of the FRO, content of conjugated dienes, and the activity of the GR/GP system were calculated using Microsoft Excel 2007.

## 3. Results and Discussion

Lipid peroxidation has been reported as a major contributor to the loss of cell function under oxidative stress conditions [13]. For example, peroxidation attack on microsomal membranes can lead to calcium release and uncontrolled activation of calcium-dependent proteases and lipases [14, 15], whereas attack on mitochondrial membranes can alter permeability and induce a disruption of cellular energetics [16]. In addition, an accumulation of lipoperoxidation products under some pathological conditions indicates the probable involvement of oxygen radicals in these disorders [17–19]. However, question concerning ROS level change under experimental toxic hepatitis development remains unclear. In this study we investigated the dynamics of free radical oxidation and the state of the GR/GP antioxidant system in liver tissue of rats during 126 h after experimental toxic hepatitis induction. This analysis detected rapid changes in functioning of the liver in response to a xenobiotic (tetrachloromethane) and traced the downstream consequences of its biotransformation.

**Figure 1:**
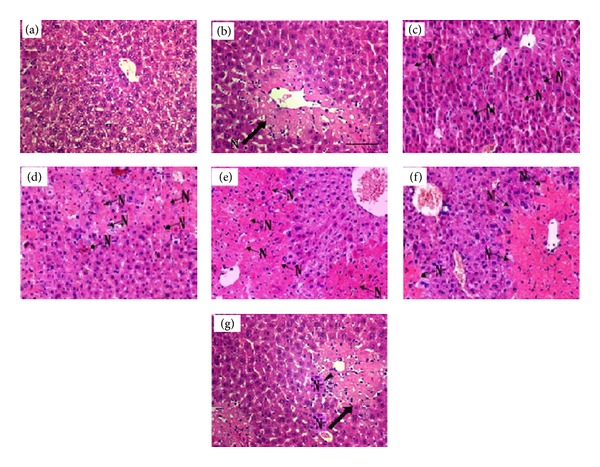
Histologic results of liver tissues stained with hematoxylin and eosin under light microscope in CCl_4_-treated rats (×200). (a) control; (b)–(g) 12, 36, 70, 96, 110, and 125 h after exposure to CCl_4_, respectively. N: necrosis.

The histologic examination of the liver sections of the control group animals showed normal histological picture of hepatic central vein and sinusoids (Figure 1(a)). The liver sections of rats treated with CCl_4_ showed prominent hepatocytes with marked vacuolization. Moreover, it was found that the most pronounced necrosis of hepatocytes, striped necrosis, and bridging necrosis were present at 96 h exposure CCl_4_, with inflammatory cells spreading around the necrotic tissue. It could also be noted that the scattering of infiltrated inflammatory cells accumulates in the liver sinusoid (Figure 1(e)). At 125-hour CCl_4_-induced hepatic injury showed a reduction in the severity of necrotic processes (Figures 1(f) and 1(g)).

**Figure 2:**
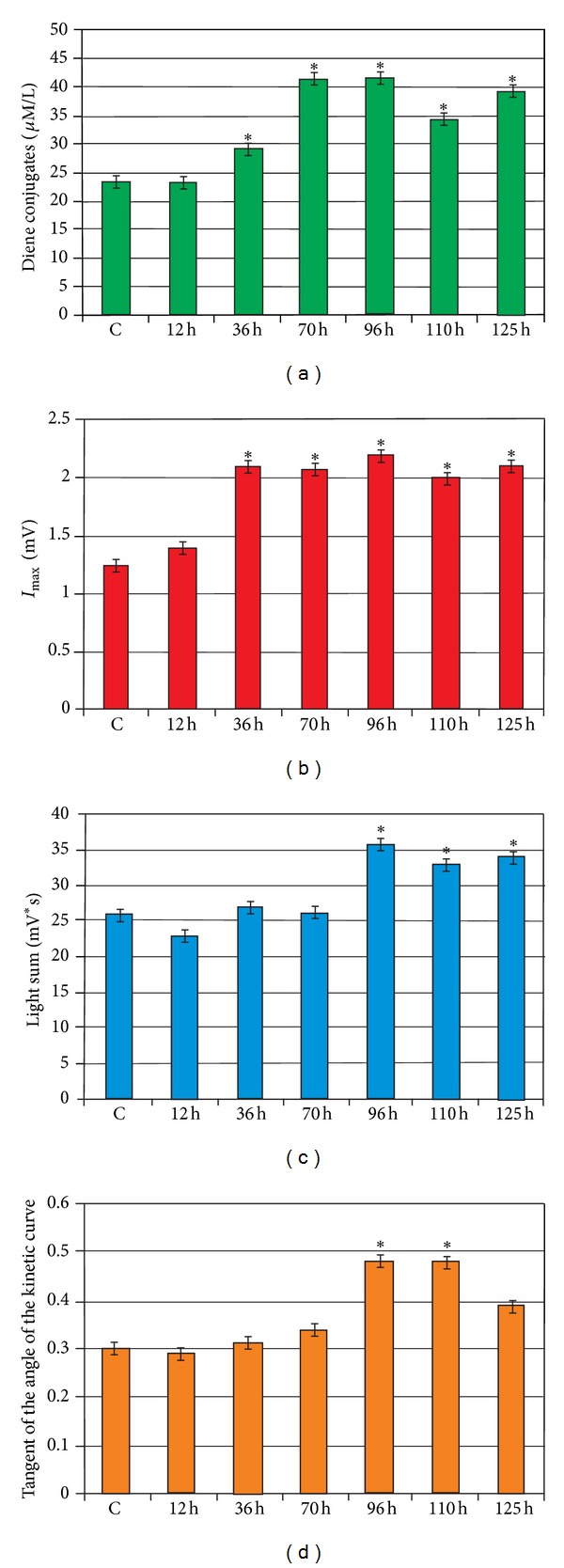
The content of diene conjugates and parameters of biochemiluminescence in rat liver during the development of experimental toxic hepatitis: (a) content of diene conjugates (umol/L). (b) Maximum flash intensity (Imax, mV). (c) Light sum, mV ∗ c. (d) Tangent of the angle of the kinetic curve. Compared with control: *P < 0.05.

The spectral characteristics of the liver lipids extracted in heptanoic phase showed an increase in optical density in the region 230–268 nm with a pronounced peak at 233 nm. These features indicate the presence of conjugated diene and lipid hydroperoxide in the studied samples. The development of liver disease increased 1.78-fold the content of diene conjugates during the first 70 h after administration of tetrachloromethane. The maximum level of diene conjugates, exceeding the reference value of 1.82 times, was observed at 96 h after administration of the toxic agent (Figure 2). The level of diene conjugates was 1.47 and 1.70 times higher than in control at 110 and 125 h of experimental toxic hepatitis development, respectively. This may suggest that activation of LPO and accumulation of lipid peroxidation products in liver tissue of the animals under induction of the xenobiotic indeed took place.

The light sum of chemiluminescence (*S*) and the maximum flash intensity (Imax), reflecting the intensity of FRO, significantly increased and reached a peak 96 h after induction of pathology (Figure 2). Compared to control values, *S* increased 2.70 times while the value of Imax increased 1.95 times. These results are in accordance with the previous studies reporting that chemiluminescence emission is induced by an oxidative stress [20]. Ninety-six h after administration of CCl_4_ and induction of liver pathology an increase in the value of the slope of the kinetic curve (tg*α*_2_) was observed. Such change indicated that the total antioxidant activity increased 2.25 times compared to the norm. This suggests that under pathological conditions an effective compensatory mechanisms reducing FRO level in the cell are necessary. The changes in GP activity during the development of experimental toxic hepatitis showed that the maximum activity was observed at 96 h after administration of tetrachloromethane. In this case, the activity per gram of fresh weight and specific activity increased 4.60 and 2.50 times, respectively.

**Figure 3:**
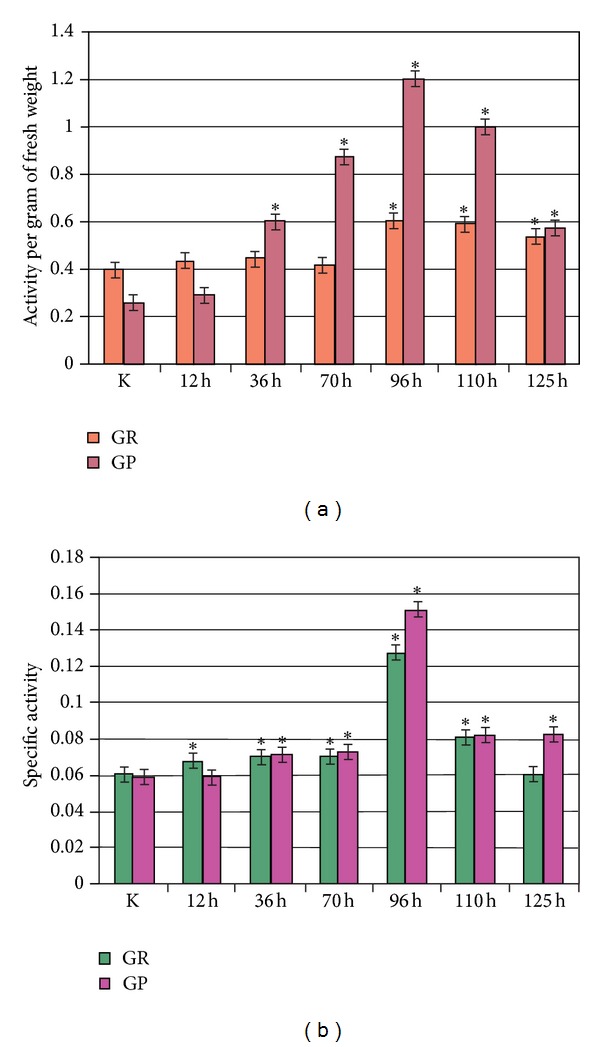
Activity of glutathione reductase and glutathione peroxidase, presented in the form of E per gram of fresh weight (a) and specific activity (b) in the liver of rats in normal conditions (K) and the dynamics of experimental toxic hepatitis. Compared with control: *P < 0.05.

The maximum activity of GR in liver of rats under experimental toxic hepatitis was recorded 96 h after the induction of pathology. The activity per gram of fresh weight and specific activity increased 1.50 and 2.10 times, respectively. After 110 h of experimental toxic hepatitis specific activity GR increased 1.30 times while specific activity of GP rose 1.40 times. Apparently, the observed changes in the activity of GP and GR (Figure 3) were a protective reaction in response to the intensification of FRO at the developmental stage of experimental toxic hepatitis, and may have resulted from either their activation or synthesis stimulation. A temporary increase in the activity of GP and GR after intoxication of rats by xenobiotics was reported previously [21].

The correlation coefficients reflecting the relationship between the GR activity and the content of diene conjugates, Imax and *S* reached the following values: 0.44, 0.50, and 0.86, respectively, while correlation coefficients between the concentration of diene conjugates, Imax, *S*, and the GP activity showed the following values: 0.96, 0.94, and 0.97. These results suggest that the intensity of FRO was correlated with the activity of the GR/GP system. However, the mechanism linking the activation of GP and GR enzymes with the intensification of FRO is still not entirely clear. To elucidate this question, the expression of GR/GP system during the experimental toxic hepatitis in rats was analyzed. Electrophoretical analysis of total RNA extracted from rat liver at 96 h of experimental toxic hepatitis demonstrated that the 28S rRNA greatly predominated over the 18S rRNA in the sample.

This observation indicates that there was no degradation of extracted RNA due to the action of ribonucleases. Optimization of the time, temperature, and concentration of primers of the polymerase chain reaction allowed for achieving high efficiency and specificity of the amplification. Sequence analysis of the PCR products showed their correspondence to the respective sequences of Gpx1 and Gsr.

**Figure 4:**
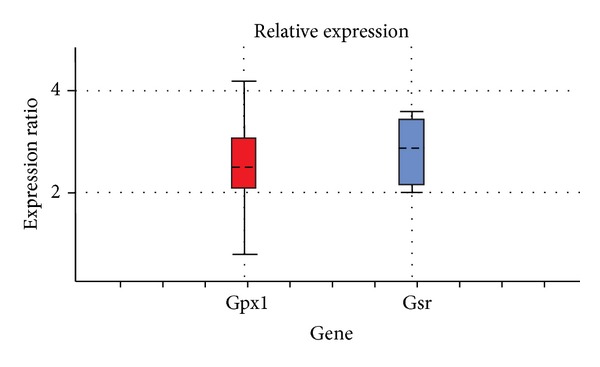
The level of Gsr and Gpx1 gene transcription in rat liver at 96 h of toxic hepatitis development, calculated using the Rest 2008 software (the arrow shows the median of statistical distribution).

Analysis of gene expression of Gpx1 and Gsr with regard to the efficiency of amplification revealed that in 96 h after the administration of the xenobiotic and induction of pathology level, these transcripts increased. The expression of Gpx1 increased 2.50 times, while Gsr 3.00 times relatively the norm (Figure 4).

**Figure 5:**
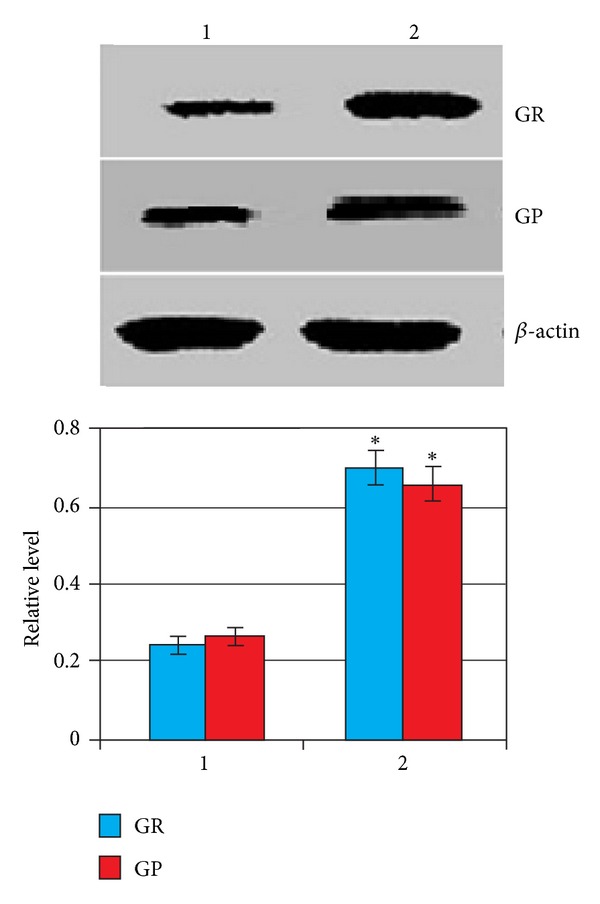
The Western blotting analysis of protein level for GR and GP, or actin in liver isolated from control group of rats (1) and CCl_4_-induced animals at 96 h after exposure (2). Compared with control: *P < 0.05.

Western blot analysis revealed that the amounts of hepatic GP and GR proteins increased considerably after CCl_4_ administration (Figure 5).

**Figure 6:**
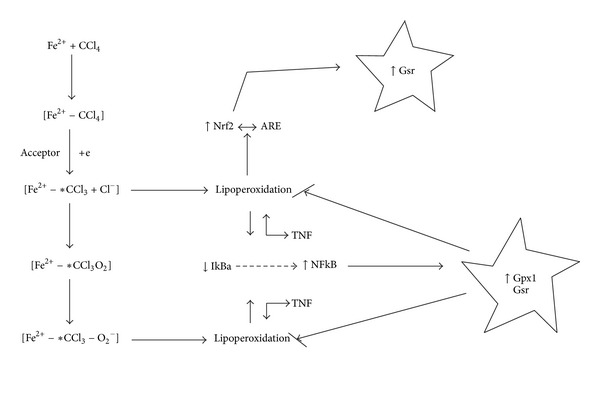
Hypothetical mechanism of activation of Gpx, Gsr by carbon tetrachloride.

The involvement of free radicals in pathogenesis of liver injury has been investigated during 125 hours development of toxic hepatitis. CCl_4_ has been extensively used as model system to study the hepatic damage [22]. We provided a set of evidence that CCl_4_-mediated oxidative stress contributed to an activation of some links of GSH dependent antioxidant system and increased diene conjugates level and parameters of biochemiluminescence. It is well established that CCl_4_ is biotransformed by P450 system to produce 3-chloromethyl free radical (Figure 6).

This leads to lipid peroxidation and finally to cell death [23, 24]. It has been reported that P450 in rat liver activates CCl_4_ and stimulates Kupffer cells for production of reactive oxygen species (ROS), such as O_2_^•−^, H_2_O_2_, and OH^•^, which damage the liver [25]. In our study the high levels of ROS may have led to an increase of diene conjugates quantity in rat liver under toxic hepatitis (Figure 2). According to a literature data, hepatic changes occurring in liver treated with carbon tetrachloride include induction of nuclear factor kappaB (NF-*κ*B) and interleukin-6 at the time of G1/S transition, induction of cyclin D1 expression associated with an increase of mitosis, and a time-dependent increase of TNF consistent with the appearance of necrosis [25–29]. It is important to notice that TNF induced oxidative stress in isolated mouse hepatocytes [26, 30]. One can anticipate that intensification of free radical processes may promote activation of GR and GP (Figure 3). As well known, NF-*κ*B might regulate Gpx and Gsr [31, 32]. Free radicals can activate NF-*κ*B by inactivation of IkBa [33]. NF-*κ*B also regulates genes involved in inflammation, apoptosis, proliferation, and regeneration in liver [31]. It could be proposed, that activation of NF-*κ*B induced expression of Gpx1 and Gsr (Figures 4 and 6). Similarly, high concentration of Gsr and Gpx1 transcripts under experimental toxic hepatitis may be connected with activation of Nrf. According to a literature data, expression of Nrf increases at oxidative stress [34, 35]. Transcription of antioxidant enzymes is regulated by antioxidant response elements (AREs). Earlier it was shown that Nrf2 (NF-E2-related factor 2) and Nrf1 are transcription factors that bind to AREs and activate these genes. Inactive Nrf2 is retained in the cytosol by an association complex with the cytoskeletal protein Keap1. Phosphorylated cytosolic Nrf2 translocates into the nucleus in response to protein kinase C activation and Map kinase pathways. In the nucleus, Nrf2 activates genes through AREs by interacting with transcription factors in the bZIP family, including CREB, ATF4, and fos or jun. Nrf2 activation of genes can be blocked by small maf proteins, including MafG and MafK [36–43]. Thus, AREs function is associated with GSR expression [44, 45]. Harvey with colleagues determined that Nrf2 regulates its target gene transcripts by directly binding to a cis-enhancer element. The analysis of the GSR promoter in silico revealed three potential AREs within 2 kb upstream of the translation start point. The sequences of all three AREs in the GSR promoter were similar to the prototypical AREs identified in Nrf2-regulated antioxidants such as NQO1 and HO-1 [44]. Most likely, ARE is used at regulation of Gsr expression under toxic hepatitis in rat liver. Based on the obtained results we postulate that one of the factors increasing activity of the GR/GP system could be the induction of synthesis of these enzymes by the xenobiotic tetrachloromethane. Overexpression of these enzymes is required to increase cellular resistance to oxidative stress, which is one of the key issues in the pathogenesis of toxic liver damage.

## 4. Conclusions

The results of this study indicate that induction of synthesis of these enzymes may be one of the factors promoting increased activity of the GR/GP system. It is possible that overexpression of these enzymes is important for increased resistance to oxidative stress, the key pathogenic factor in various diseases.

## Highlights

- Glutathione peroxidase (GP) and glutathione reductase (GR) system plays key role in organism defense under toxic hepatitis.
- The Gpx1 and Gsr enzyme's genes expression was detected at the action of tetrachloromethane.
- The enzyme's expression can be a mechanism of tissue tolerance to oxidative stress.
- This might be the first sign of the role of xenobiotics in the overexpression of the GP/GR system enzymes.

## Figures and Tables

**Table 1:** Primers of genes Gpx1, Gsr, *β*Act, Alb, and L3 used for real-time PCR.

Primer	Sequence	Sequences source in NCBI	Efficiency amplification, %	Generally Tm, °C
Gpx1 (forward) Gpx1 (reverse)	AGTTCGGACATCAGGAGAATGGCA TCACCATTCACCTCGCACTTCTCA	NM_030826.3	86.3	68
Gsr (forward) Gsr (reverse)	GGAAGTCAACGGGAAGAAGTTCACTG CAATGTAACCGGCACCCACAATAAC	NM_053906.1	91.28	64
*β*Act (forward) *β*Act (reverse)	TGTTTGAGACCTTCAACACC TAGGAGCCAGGGCAGTAATC	NM_031144.2	94.9	63
Alb (forward) Alb (reverse)	GCAAACTGCAGGCTTGCTGTGATA AAGTCAGCAGCTATTGAGGGCAGA	NM_134326	98.03	60
L3 (forward) L3 (reverse)	GCATTCATGGGACCGCTCAA AGCTGCACAAAGTACTCCTGGCAT	NM_198753	89.23	68

## Abbreviations

BHL: Biochemiluminesce
NF-*κ*B: Nuclear factor kappa B
GP/GR: Glutathione peroxidase/glutathione reductase system
RT-PCR: Real-time polymerase chain reaction
Alb: Albumin
Ct: Threshold cycle
Gpx1: Gene of glutathione peroxidase 1
Gsr: Gene of glutathione reductase
L3: Ribosomal protein
REF: Reference gene
REST: Computer software for calculating the level of gene expression
Tm: Melting temperature of primers
*β*Act: *β*-actin
TNF: Tumor necrosis factor
STAT3: Signal transducer and activator of transcription 3
ROS: Reactive oxygen species
TGF-beta1: Transforming growth factor beta 1
IkBa: Proteins inhibitor of NF-*κ*B
ARE: Antioxidant response elements
bZIP: G-box binding factor
CREB: cAMP response element-binding protein
ATF4: Anti-Activating Transcription Factor 4
Maf: Transcription factor
NQO1: NAD(P)H dehydrogenase, quinone 1
HO-1: Heme oxygenase-1
MafG: v-maf musculoaponeuroticfibrosarcoma oncogene
MafK: v-maf musculoaponeuroticfibrosarcoma oncogene homolog K
FR: Free radical
FRO: Free radical oxidation.
